# Risk of Selected Cardiovascular Toxicities in Patients With Cancer Treated With MEK Inhibitors: A Comparative Systematic Review and Meta-Analysis

**DOI:** 10.1200/JGO.2015.000802

**Published:** 2015-11-25

**Authors:** Omar Abdel-Rahman, Hesham ElHalawani, Hoda Ahmed

**Affiliations:** Omar Abdel-Rahman and Hesham ElHalawani, Ain Shams University; and Hoda Ahmed, Nasser Institute, Cairo, Egypt.

## Abstract

**Purpose:**

We conducted a literature-based meta-analysis of the risk of cardiovascular toxicities associated with MEK inhibitors.

**Methods:**

Eligible trials included randomized phase II and III trials of patients with cancer who were given a mitogen activated protein (MAP)/extracellular signal-regulated kinase (ERK) kinase (MEK) inhibitor (trametinib, selumetinib, or cobimetinib) and that described events of hypertension and decreased ejection fraction.

**Results:**

Our search strategy yielded 300 potentially relevant citations from PubMed/MEDLINE, Google Scholar, and Cochrane Central Register of Controlled Trials. After ineligible studies were excluded, a total of 10 clinical trials were considered eligible for the meta-analysis. The relative risk for all grades of hypertension was 1.54 (95% CI, 1.02 to 2.32; *P* = .05), 1.85 (95% CI, 1.01 to 3.40; *P* = .05) for high-grade hypertension, and 4.92 (95% CI, 2.93 to 8.25; *P* < .001) for decreased ejection fraction. Subgroup analysis revealed no difference between trametinib and selumetinib for risk of hypertension.

**Conclusion:**

Our meta-analysis demonstrated that MEK inhibitor–based treatment is associated with an increased risk of all-grade and high-grade hypertension and asymptomatic decrease in ejection fraction. Clinicians should be aware of this risk and perform regular assessment.

## INTRODUCTION

The mitogen activated protein (MAP)/extracellular signal-regulated kinase (ERK) kinase (MEK) inhibitors are an emerging group of active anticancer agents that have recently been presented as reliable alternatives in the management of a variety of solid tumors, including advanced melanoma and advanced non–small-cell lung cancer (NSCLC).^1–4^ Trametinib, selumetinib, and cobimetinib are the most common clinically studied agents in this group, and they are essentially tyrosine kinase inhibitors that have shown a wide spectrum of preclinical and clinical activity against many solid tumors.^5^ Trametinib has been approved for the treatment of BRAF-mutant advanced melanoma.^6^ In addition, both trametinib and selumetinib have been assessed in KRAS-mutant NSCLC.^7,8^ Several ongoing phase II and III studies are assessing the three agents in other solid tumor indications.

The peculiar mechanism of action of MEK inhibitors has been linked to a characteristic pattern of mechanism-driven adverse events, including GI, cutaneous, cardiac, and vascular events.^6,9,10^ This may be directly related to the inhibition of the mitogen-activated protein kinase (MAPK) pathway. However, there has been no systematic attempt to synthesize the data regarding cardiovascular (CV) toxicities of these agents, and the overall risk of CV toxicities induced by these agents needs to be further assessed. The range of CV toxicities assessed in our analysis includes hypertension and asymptomatic decline in ejection fraction (EF).

### Objective of the Meta-Analysis

We conducted a meta-analysis of randomized clinical trials (RCTs) to determine the overall risk of selected CV toxicities in patients with cancer who were treated with MEK inhibitors.

## METHODS

### Data Source

We conducted a detailed review of MEDLINE and Google Scholar databases from 2010 to June 2015 by using “selumetinib” OR “trametinib” OR “cobimetinib” OR “MEK inhibitor” as search keywords. Our search was restricted to RCTs that recruited patients with cancer and were published in English. Trials were systemically reviewed according to the Preferred Reporting Items for Systematic Reviews and Meta-Analyses statement.^11^ Quality of the included studies was assessed by using the Jadad scale.^12^

### Study Selection

Clinical trials that met the following criteria were included: the study was a randomized phase II or III study in patients with cancer, participants were randomly assigned to treatment with MEK inhibitor–based therapy versus controls, and data regarding sample size and events were available for hypertension and decreased EF.

Independent reviewers (H.E. and H.A.) audited reports that incorporated the search terms initially by their titles and abstracts for relevance; later, the full texts were scanned for eligibility.

### Data Extraction and Clinical End Points

Two review authors (O.A.-R. and H.E.) independently extracted the data. A checklist of data to be extracted from each study included the name of the first author, the trial phase, the type of cancer, the treatment regimens in different arms, and the number of each type of adverse event. Any discrepancies between the authors were resolved by consulting a third author. Most of the included trials used the Common Terminology Criteria for Adverse Events version 4.0 for grading the relevant adverse events.

### Data Analysis

The principal measure analyzed was relative risk (RR) and its corresponding 95% CIs for all-grade (grades 1 to 4) and high-grade (grades 3 to 4) selected CV toxicities. Cochrane's *Q* statistic was applied to assess statistical heterogeneity in adverse events between trials, and inconsistency was quantified by using the *I*^2^ statistic. A *P* value threshold of .1 was regarded as a threshold between homogeneous and heterogeneous calculation, and *P* < .1 was considered suggestive of heterogeneity. Whenever no substantial heterogeneity existed, the pooled estimate was determined on the basis of the fixed-effects model. Along the same lines, the pooled estimate was calculated on the basis of the random-effects model by using the DerSimonian method whenever a substantial heterogeneity prevailed.^13,14^ Data analyses were performed by using Review Manager 5.3 (Nordic Cochrane Centre, Copenhagen, Denmark).

## RESULTS

### Search Outcome

**Figure 1:**
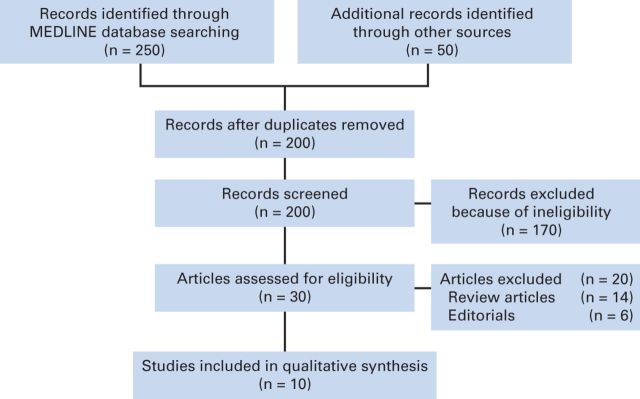
Flowchart of study selection procedure.

Our search yielded 300 potentially relevant citations about MEK inhibitors from the searched databases. The details for the study inclusion/exclusion procedure are illustrated in Figure 1.

A total of 10 RCTs were considered eligible for the analysis,^8,15–23^ including five phase III trials^15,16,19,21,23^ and five phase II trials.^8,17,18,20,22^ Seven studies assessed treatment for advanced melanoma,^15–17, 19, 21–23^ one study assessed treatment for advanced NSCLC,^8^ one study assessed treatment for advanced pancreatic carcinoma,^20^ and one study evaluated treatment for advanced breast cancer.^18^ Six studies involved trametinib,^8,15,16,20,21,23^ three studies evaluated selumetinib,^17,18,22^ and one study investigated cobimetinib.^19^ Jadad quality score for the included studies ranged from 3 to 5 (Table 1).

**Table 1:** Jadad Quality Score for the Included Studies

Study	Year	Randomization	Blinding	Account of All Patients	Total Score
Flaherty et al^15^	2012	2	0	1	3
Robert et al^23^	2015	2	0	1	3
Long et al^21^	2014	2	2	1	5
Flaherty et al^16^	2012	2	0	1	3
Infante et al^20^	2014	2	2	1	5
Blumenschein et al^8^	2015	2	0	1	3
Robert et al^22^	2013	2	2	1	5
Kirkwood et al^17^	2012	2	0	1	3
Zaman et al^18^	2015	2	2	1	5
Larkin et al^19^	2014	2	1	1	4

### Patient Characteristics

A total of 2,704 patients were included in this analysis. Most patients had an Eastern Cooperative Oncology Group performance score between 0 and 2 and competent liver, kidney, and bone marrow function. Overall, age and sex were equally distributed in the majority of studies. Several other prognostic variables were reported in some studies and were generally comparable among randomized groups. The baseline patient characteristics and the number of adverse events in each trial are described in Table 2.

**Table 2:** Baseline Patient Characteristics and Number of Adverse Events in Each Trial

Study	Year	Quality (Jadad score)	Study Type	Cancer Type	Treatment Regimen (No. of patients)	Baseline Patient Characteristics	Indication	Decreased Ejection Fraction	Hypertension	
									All-Grade	High-Grade
Trametinib studies										
Flaherty et al^16^	2012	3 (open-label)	Phase III RCT	Melanoma	Arm A: trametinib 2 mg orally once per day (211 patients) Arm B: intravenous chemotherapy consisting of either dacarbazine 1,000 mg/m^2^ or paclitaxel 175 mg/m^2^ once every 3 weeks (99 patients)	Median age: 55 (23-85) *v* 54 (21-77) Male sex: 120 (56%) *v* 53 (49%) White race: 214 (100%) *v* 108 (100%)	First-line therapy for cutaneous advanced or metastatic melanoma (stage IIIC or IV) with a BRAF V600 mutation-positive tumor sample	11 patients (5.2%) in the trametinib arm only	32 (15%) *v* 7 (7%)	26 (12%) *v* 3 (3%)
Robert et al^23^	2015	3 (open-label)	Phase III RCT	Melanoma	Arm A: combination of dabrafenib 150 mg twice per day and trametinib 2 mg once per day (350 patients) Arm B: vemurafenib 960 mg orally twice per day (349 patients)	Median age: 55 (18-91) *v* 54 (18-88) Male sex: 208 (59%) *v* 180 (51%)	First-line therapy for cutaneous advanced or metastatic melanoma (stage IIIC or stage IV) with a BRAF V600 mutation-positive tumor sample	29 patients (8%) in arm A only	92 (26.3%) *v* 85 (24.4%)	N/R
Long et al^21^	2014	5	Phase III RCT	Melanoma	Arm A: combination of dabrafenib 150 mg orally twice per day and trametinib 2 mg orally once per day (209 patients) Arm B: dabrafenib and placebo (211 patients)	Median age: 55 (22-89) *v* 56.5 (22-86) Male sex: 111 (53%) *v* 114 (54%)	First-line therapy for cutaneous advanced or metastatic melanoma (stage IIIC or stage IV) with a BRAF V600 mutation-positive tumor sample	9 (4%) *v* 5 (2%)	46 (22%) *v* 29 (14%)	8 (4%) *v* 10 (5%)
Flaherty et al^15^	2012	3 (open-label)	Phase III RCT	Melanoma	Arm A: dabrafenib monotherapy 150 mg orally once per day (55 patients) Arm B: combination of dabrafenib 150 mg orally twice per day and trametinib 1 mg orally once per day (54 patients) Arm C: combination of dabrafenib 150 mg orally twice per day and trametinib 2 mg orally once per day (55 patients)	Median age: 50 (18-82) *v* 49 (23-85) *v* 58 (27-79) Male sex: 29 (54%) *v* 30 (56%) *v* 34 (63%)	First-line therapy for patients with BRAF-mutant metastatic melanoma	0 *v* 2 (4%) *v* 5 (9%)	2 (4%) *v* 2 (4%) *v* 5 (9%)	1 (2%) patient in arm C only
Infante et al^20^	2014	5	Phase II RCT	Pancreas	Arm A: trametinib 2 mg per day plus intravenous gemcitabine 1,000 mg/m^2^ once per week for 8 weeks, then days 1, 8, and 15 of 28-day cycles (80 patients) Arm B: placebo plus intravenous gemcitabine 1,000 mg/m^2^ once per week for 8 weeks, then days 1, 8, and 15 of 28-day cycles (80 patients)	Median age: 64 (42-85) *v* 63.5 (41-82) Age group ≥ 65: 39 (49%) *v* 34 (43%) Male sex: 39 (49%) *v* 46 (58%) White/European heritage: 50 (63%) *v* 59 (74%)	First-line therapy for untreated metastatic adenocarcinoma of the pancreas	7 (8.8%) *v* 2 (2.5%)	2 (2.5%) *v* 6 (7.5%)	N/R
Blumenschein et al^8^	2015	3 (open-label)	Phase II RCT	NSCLC	Arm A: trametinib 2 mg orally once per day (87 patients) Arm B: docetaxel 75 mg/m^2^ intravenously once every 3 weeks (43 patients)	Median age: 63 (40-79) *v* 63 (34-79) Male sex: 46 (53%) *v* 23 (53%) White race: 74 (87%) *v* 34 (79%) Smoking status: current: 13 (15%) *v* 13 (30%); former: 67 (78%) *v* 23 (53%)	Second-line therapy for histologically confirmed *KRAS*-mutant NSCLC previously treated with one prior platinum-based chemotherapy	5 patients (5.8%) in arm A only	13 (15%) *v* 1 (2%)	8 (9%) grade 3 events in arm A only
Selumetinib studies										
Robert et al^22^	2013	5	Phase II RCT	Melanoma	Arm A: intravenous dacarbazine 1,000 mg/m^2^ on day 1 of a 21-day cycle plus oral selumetinib 75 mg twice per day on a 21-day cycle (44 patients) Arm B: intravenous dacarbazine 1,000 mg/m^2^ on day 1 of a 21-day cycle plus placebo (45 patients)	Median age: 57 (48-69) *v* 52 (40-65) Male sex: 22 (49%) *v* 28 (61%)	First-line treatment for *BRAF*-mutant metastatic melanoma	7 (16%) *v* 1 (2%)	N/R	
Kirkwood et al^17^	2012	3 (open-label)	Phase II RCT	Melanoma	Arm A: oral selumetinib 100 mg twice per day on 28-day cycles (99 patients) Arm B: oral temozolomide 200 mg/m^2^ per day for 5 days, then 23 days off treatment (95 patients)	Mean age: 57.1 (20-84) *v* 57 (28-84) Male sex: 55 (52.9%) *v* 65 (67.7%) White race: 99 (95.2%) *v* 91 (94.8%)	Chemotherapy-naive patients with unresectable stage III to IV melanoma	N/R	8 (8.1%) *v* 2 (2.1%)	
Zaman et al^18^	2015	5	Phase II RCT	Breast	Arm A: fulvestrant 500 mg intramuscularly on days 1, 15, and 29 of cycle 1 and then every 28 ± 3 days plus selumetinib 75 mg orally twice per day (23 patients) Arm B: fulvestrant 500 mg intramuscularly on days 1, 15, and 29 of cycle 1 and then every 28 ± 3 days plus placebo (22 patients)	Median age: 66 (40-79) *v* 69 (46-79); all included patients were postmenopausal women	Second-line treatment in postmenopausal women with advanced-stage endocrine sensitive breast cancer	N/R	All-grade: 5 (23%) *v* 5 (24%) High-grade: 1 (4.3%) *v* 2 (9.1%)	
Cobimetinib studies										
Larkin et al^19^	2014	4 (blinding method was not described)	Phase III RCT	Melanoma	Arm A: oral vemurafenib 960 mg twice per day together with cobimetinib 60 mg once per day for 21 days, followed by 7 days off treatment (254 patients) Arm B: oral vemurafenib 960 mg twice per day together with placebo (239 patients)	Median age: 56 (23-88) *v* 55 (25-85) Male sex: 146 (59%) *v* 140 (56%) White race: 227 (92%) *v* 235 (95%)	First-line therapy for cutaneous advanced or metastatic melanoma (stage IIIC or IV) with a BRAF V600 mutation-positive tumor sample	19 (7.5%) *v* 7 (2.9%)	N/R	

NOTE: Age is provided in years; range is in following parentheses.

Abbreviations: N/R, not reported; NSCLC, non–small-cell lung cancer; RCT, randomized controlled trial.

### Overall Incidence of Relevant Adverse Events

All-grade hypertension ranging from 2.5% to 15% was reported in seven studies; high-grade hypertension ranging from 2% to 12% was reported in only four trametinib studies. Decreased EF ranging from 4% to 23% was reported in nine studies, whereas clinical congestive heart failure (CHF) was reported in only one trametinib study.^16^

### RR of All-Grade Relevant Adverse Events

A meta-analysis of the RR of all-grade adverse events was performed on the RCTs that contained direct comparison between MEK inhibitors and control treatment. The RR for all-grade hypertension was 1.54 (95% CI, 1.02 to 2.32; *P* = .05), 1.85 (95% CI, 1.01 to 3.40; *P* = .05) for high-grade hypertension, and 4.92 (95% CI, 2.93 to 8.25; *P* < .001) for decreased EF (Fig 2A, Fig 2B, and Fig 3). Because CHF was reported in only one study, RR could not be assessed for this toxicity.

**Figure 2:**
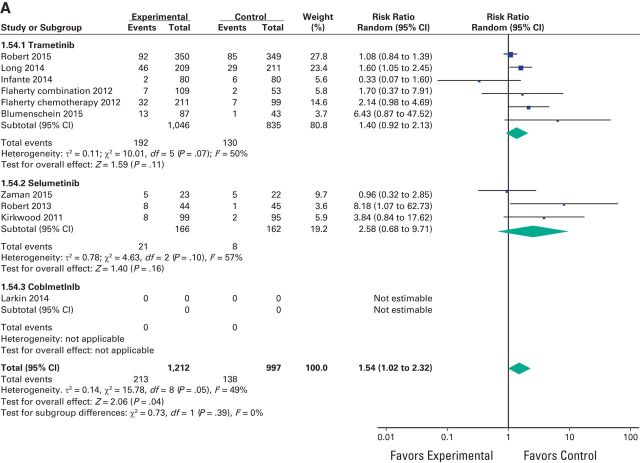
Forest plots of risk ratio of (A) all-grade hypertension associated with MEK inhibitors versus control and (B) high-grade hypertension associated with MEK inhibitors versus control; the size of squares corresponds to the weight of the study in the meta-analysis.

**Figure 2:**
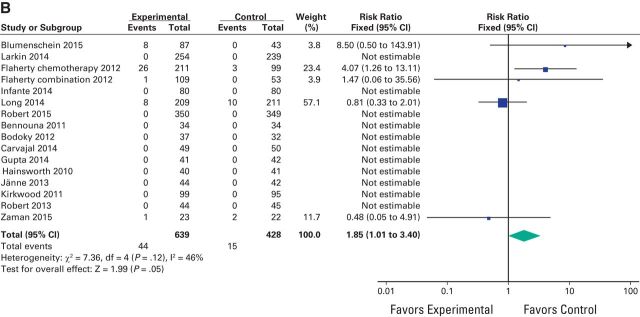
Continued

**Figure 3:**
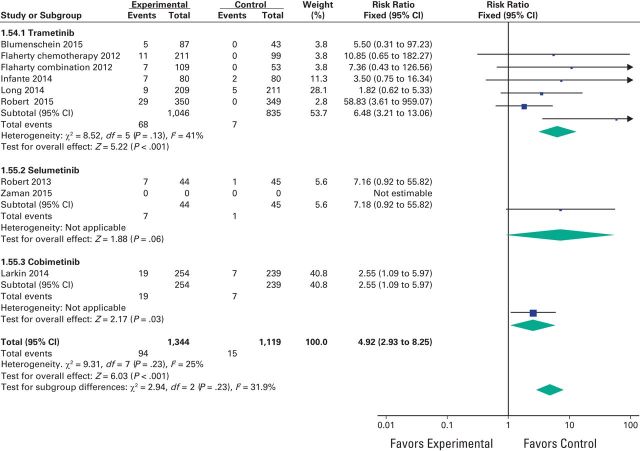
Forest plots of risk ratio of decreased ejection fraction associated with MEK inhibitors versus control.

**Figure 4:**
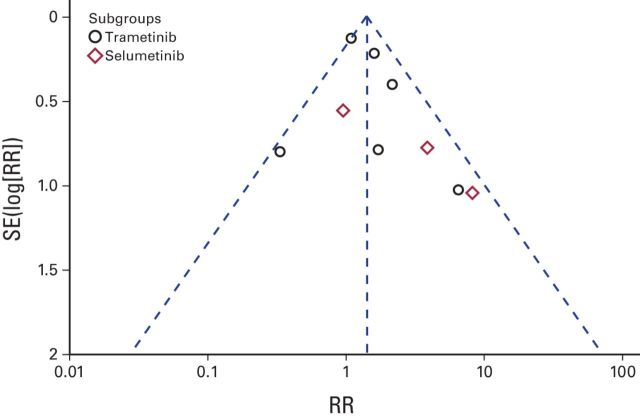
Funnel plot for publication bias for all-grade hypertension. RR, risk ratio.

**Figure 5:**
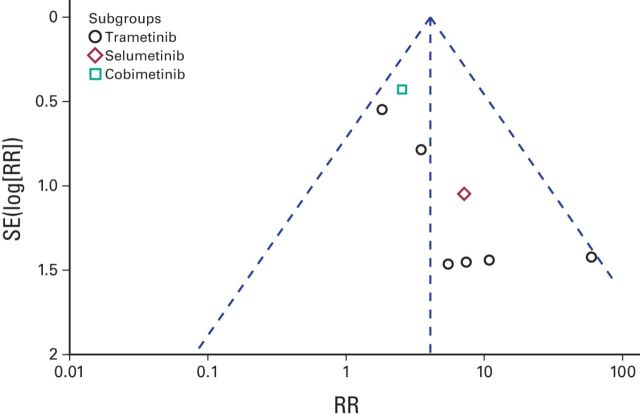
Funnel plot for publication bias for decreased ejection fraction. RR, risk ratio.

Thus, patients treated with MEK inhibitors have an increased risk of all-grade and high-grade hypertension and asymptomatic decrease in EF. The fixed-effects model was used for high-grade hypertension and decreased EF, and the random-effects model was used for all-grade hypertension. Funnel plot analysis gave the impression of a publication bias (only three studies were within the left side of the inverted V shown in Figure 4 and Figure 5). We have used simple sensitivity tests to address this point.

### Subgroup Analysis According to the Type of Agent Used

We conducted subgroup analyses for the risk of hypertension and decreased EF according to the type of agent used but we did not find a statistically significant difference between these subgroups for hypertension (*P* = .39) despite a nearly doubled RR for selumetinib (2.58) versus 1.40 for trametinib. For decreased EF, a subgroup analysis could not be performed because selumetinib and cobimetinib were represented by only one study each (Fig 2A and Fig 3).

### Sensitivity Analyses

The RR of all-grade and high-grade hypertension was analyzed after excluding the study by Zaman et al^18^ because patients with breast cancer may be at a higher risk of cardiotoxicity as a result of previous doxorubicin; however, the risk of both all-grade and high-grade hypertension was still higher even after excluding this study. The RR for all-grade hypertension was 1.64 (95% CI, 1.04 to 2.59; *P* = .03), and it was 2.04 (95% CI, 1.08 to 3.86; *P* = .03) for high-grade hypertension.

For decreased EF, a second assessment of the RR was performed by using a random-effects model instead of a fixed-effects model to obtain more conservative results, and again, it revealed an increased RR of 3.88 (95% CI, 1.93 to 7.82; *P* < .001).

## DISCUSSION

To the best of our knowledge, this is the most updated meta-analysis that provides an evaluation of the incidence and risk of selected CV toxicities in patients with cancer who were treated with MEK inhibitors. Our analysis of data demonstrated an increased risk of all-grade and high-grade hypertension and asymptomatic decrease in EF with MEK inhibitor–based treatment compared with controls. The three MEK inhibitors evaluated in our analysis were trametinib, selumetinib, and cobimetinib. Subgroup analysis revealed no difference between trametinib and selumetinib for risk of hypertension.

The MAPK pathway (also known as the RAS-RAF-MEK-ERK pathway) is frequently mutated in many solid malignancies. Activation of this pathway regulates a range of biologic processes including proliferation and survival.^24^

CV adverse events are considered an important cause for treatment interruption or discontinuation in studies of MEK inhibitors; however, we do not currently have reliable methods to predict patients at highest risk, and thus regular monitoring of patients receiving these agents should be considered standard practice.

CV toxicities (particularly hypertension and left ventricular dysfunction) have been reported with many other cytotoxic and targeted therapeutics used in cancer management, and likewise they have been linked to dose reduction and/or interruption.^25,26^

Several pathogenetic mechanisms have been proposed to explain the development of cardiac dysfunction secondary to MAPK pathway targeting. For example, in a neonatal rat myocyte model, treatment of myocytes with sorafenib caused dose-dependent damage at therapeutically relevant concentrations. This has been ascribed to the inhibition of RAF1 and BRAF kinases by sorafenib.^27^ Similar results were also found by sunitinib in a neonatal rat model (although the main mechanism of action of sunitinib is on the vascular endothelial growth factor pathway rather than on the MAPK pathway).^28^

Contrary to the cardiotoxic effects of MEK inhibitors in patients with cancer discussed earlier, some preclinical studies have shown an interesting cardioprotective effect of these agents in mice with mutations in the A-type nuclear lamins (LMNA) gene, which leads to a dilated cardiomyopathy.^29^ Moreover, a synergistic cardioprotective effect has been shown in combination with angiotensin II–converting enzyme inhibitors in mice diagnosed with the same condition.^30^ How to exploit these findings in the best way to ameliorate the potential cardiotoxicity of MEK inhibitors should be an active area of research, and this remains to be clarified.

Several agents have been shown to have a cardioprotective effect in the setting of anticancer agents. In particular, angiotensin-converting enzyme inhibitors and angiotensin-receptor blockers have been studied with anthracyclines and trastuzumab.^31^ Whether these agents play a protective role with MEK inhibitors (especially because they are also potent antihypertensives) remains to be evaluated and clarified.

Earlier detection of cardiac toxicity secondary to anticancer drugs has been an interesting target for researchers for a long time. Several radiologic and laboratory biomarkers have been proposed as good candidates to achieve this. For example, by using a murine model of bevacizumab- and sunitinib-mediated cardiotoxicity, Bordun et al^32^ investigated whether cardiac biomarkers and/or tissue velocity imaging by using echocardiography can detect early changes in cardiac function before a decrease in EF can be identified. They found that although serum cardiac biomarkers were not predictive of early left ventricular systolic dysfunction, tissue velocity imaging confirmed early left ventricular systolic dysfunction 5 days before the echocardiographic documentation of decreased EF.

Despite the high risk of decrease in EF with these agents, clinical CHF was not reported in most of the studies. This may be ascribed to the fact that in the setting of controlled clinical trials, meticulous follow-up with early referral to appropriate cardio-oncology services to institute proper treatments prevents deterioration into frank, clinically apparent, heart failure.

Regarding hypertension, we suggest that patients receiving MEK inhibitors should have baseline measurement of blood pressure and then regular monitoring throughout the treatment period.

A practical approach to the management of hypertension induced by these agents starts with proper grading, and we recommend the use of the three-grade system commonly used with antiangiogenic drugs. With grade I hypertension, which is defined as an asymptomatic increase to > 150/100 mmHg in a previously normal patient, no intervention is needed. With grade 2 hypertension, which is defined as a symptomatic increase to > 150/100 mmHg in a previously normal patient, initiating an antihypertensive (eg, calcium channel blockers or angiotensin-converting-enzyme inhibitors) is indicated. Moreover, dose reduction and/or discontinuation should be considered until proper control of blood pressure is achieved. For grade 3 hypertension, the drug should be stopped, with subsequent dose reduction on control of the blood pressure, or the drug may be permanently discontinued if satisfactory control of blood pressure has not been achieved.

Other relevant forms of CV toxicities which may be underreported in clinical trials include QTc prolongation. Particular care and caution has to be exercised with this toxicity because of the potential of rapidly degenerating into ventricular tachycardia with potential fatal consequences.^33^

### Weaknesses of Our Meta-Analysis

Several limitations in our analysis have to be mentioned, most importantly, the lack of homogeneity with regard to drugs used. This has been manifested in the wide variation of some event rates (eg, hypertension was not reported in the Larkin et al^19^ study compared with 15% all-grade hypertension and 12% high-grade hypertension in the Flaherty et al study).^16^ Subgroup analysis has been conducted to overcome this issue and to provide deeper understanding of the differences between individual MEK inhibitors in different CV risks. Another weakness has been evidence of publication bias in the funnel plots. We have tried to do simple sensitivity tests to overcome this issue.

In conclusion, our meta-analysis has demonstrated an increased risk of all-grade and high-grade hypertension and subclinical decreased EF with MEK inhibitor–based treatment compared with control. Clinicians should be aware of this risk and perform regular follow-up for such toxicities.
